# Mixing and mingling in visual working memory: Inter-item competition is feature-specific during encoding and feature-general during maintenance

**DOI:** 10.3758/s13414-024-02933-3

**Published:** 2024-08-12

**Authors:** Janna W. Wennberg, John T. Serences

**Affiliations:** 1 Department of Psychology, University of California, San Diego, La Jolla, CA USA; 2 Neuroscience Graduate Program, University of California, San Diego, La Jolla, CA USA

**Keywords:** Visual working memory, Working memory interference, Sensory recruitment hypothesis

## Abstract

Visual working memory (WM) is a central cognitive ability but is capacity-limited due to competition between remembered items. Understanding whether inter-item competition depends on the similarity of the features being remembered has important implications for determining if competition occurs in sensory or post-sensory stages of processing. Experiment 1 compared the precision of WM across homogeneous displays, where items belonged to the same feature type (e.g., colorful circles), and heterogeneous displays (e.g., colorful circles and oriented bars). Performance was better for heterogeneous displays, suggesting a feature-specific component of interference. However, Experiment 2 used a retro-cueing task to isolate encoding from online maintenance and revealed that inter-item competition during storage was not feature-specific. The data support recent models of WM in which inter-item interference – and hence capacity limits in WM – occurs in higher-order structures that receive convergent input from a diverse array of feature-specific representations.

## Introduction

Visual WM, the ability to hold visual information “in mind,” mediates many behaviors and is often disrupted in developmental and psychiatric disorders such as attention-deficit hyperactivity disorder (ADHD), Parkinson’s disease, depression, and schizophrenia (Gold & Luck, 2023; Schecklmann et al., 2011). A critical feature of visual WM is that it has limited capacity: most people cannot precisely remember details about more than three or four items (Adam et al., 2017; Alvarez & Cavanagh, 2004; Cowan, 2001; Luck & Vogel, 1997; Ma et al., 2014). To date, these limitations can best be explained by *inter-item interference,* where multiple items in WM compete for limited resources (Bays, 2014; Lewis-Peacock & Norman, 2014; Oberauer & Lin, 2017). Indeed, there are often distortions of individual items in memory such that items are attracted towards or repelled from other items, highlighting the intermingling between representations (Bae & Luck, 2017; Chunharas et al., 2022; Lively et al., 2021; Scotti et al., 2021).

Many models of flexible information storage explicitly or implicitly suggest that inter-item interference arises due to competition between sensory representations, which is consistent with *sensory recruitment,* or a role for sensory neurons that encode specific features in supporting high-fidelity WM for those features (Harrison & Tong, 2009; Serences et al., 2009; for reviews, see Adam et al., 2022; D’Esposito & Postle, 2015). Accordingly, behavioral studies generally suggest that competition is mediated by feature similarity (Schurgin et al., 2020), in line with the idea that interference is at least partially due to competing populations of feature-selective neurons in early visual cortex.

Other sensory recruitment models, however, assume that memories are maintained in a sensory-like format, but that competition occurs in higher-order areas where projections from sensory areas converge (Bouchacourt & Buschman, 2019; Swan & Wyble, 2014). For example, Bouchacourt and Buschman (2019) built a two-layer, feedforward spiking neural network where items were encoded in feature-selective sensory layers. These sensory neurons then sent converging random projections to a second layer, where neurons exhibited high-dimensional tuning for multiple features. Critically, inter-item interference occurs in the second layer because converging inputs from multiple sensory networks create destructive interference when too many items are simultaneously stored. Thus, this class of model suggests that interference is *feature-general* rather than *feature-specific* (i.e., competition is only determined by overall memory load, not by inter-item similarity). These neural models are thus generally consistent with the object file hypothesis, where WM recruits an object-based, content-independent “pointer” to store and update information about an object held in mind (Pylyshyn, 1989). Empirical work using contralateral delay activity (Luria & Vogel, 2011), multivariate analysis of electroencephalogram (EEG) data (Thyer et al., 2022), and computational modeling of whole report WM tasks (Ngiam et al., 2024) supports the existence of such a feature-general system.

The question of feature-specific and feature-general interference has been addressed in work about memory for conjunction objects. Some studies found that WM performance in a change-detection task is comparable when participants are holding in mind all features on an object compared to a single feature (Luck & Vogel, 1997), suggesting that the number of items – and not the specific visual features being stored – determines interference. However, work by Fougnie et al. (2010) suggests that when high mnemonic precision was required of participants – through a continuous-report task or a change-detection task with high target-lure similarity – adding features to objects resulted in reduced memory precision. Fougnie and Alvarez (2011) buttressed these findings when they used a continuous-report task with colorful, oriented objects and observed an independence of color and orientation report errors: one feature could be forgotten entirely, while the other was still recalled with relatively high precision. Critically, this independence was not observed for features that likely have highly overlapping neural codes, such as the length and width of objects. Taken together, these findings suggest that while there is an overall object-based benefit in visual WM, feature-specific content nevertheless influences performance (Fougnie et al., 2013).

In addition to objects composed of simple visual features like orientation and color, prior research using real-world objects has also found mixed-category benefits that are consistent with feature-specific interference in visual WM. Notably, Cohen et al. (2014) found that participants could remember more objects when they were from more than one category (e.g., faces and scenes) compared to when they were from one category (e.g., faces and faces). A follow-up neuroimaging experiment revealed that the size of the mixed-category benefit on a given trial was predicted by the degree of neural separability between categories (e.g., faces and scenes are processed in different neural populations; therefore, there is less cross-category competition) (Avital-Cohen & Gronau, 2021; Cohen et al., 2014; but see Jiang et al., 2016; Mruczek et al., 2019). The mixed-category benefit overall has been replicated with simple visual features such as color, orientation, luminance, and motion (Cai et al., 2022; Gosseries et al., 2018).

The goal of the current study was to evaluate interference within and between different feature spaces (i.e., feature-general or feature-specific interference) during encoding and, importantly, during memory maintenance. In Experiment 1, we compared performance on trials with *homogeneous* displays with the same types of features (e.g., a display of colorful circles) and *heterogeneous* displays with more than one type of feature (e.g., a display of colored circles and oriented bars). If inter-item interference is driven by a feature-specific component, memory precision for heterogeneous displays should be higher than memory precision for homogeneous displays. In contrast, if inter-item competition occurs in unspecialized networks during later stages of visual processing, then we should observe comparable memory performance when remembering heterogeneous displays and homogenous displays. In Experiment 2, we controlled for feature-similarity during encoding and used retro-cues to assess whether any feature-specific interference occurred during active, online maintenance of the memoranda. Together, the studies suggest that feature-specific interference occurs during encoding but not during maintenance, consistent with models positing that interference in WM happens after item-specific sensory information converges in a common, more general purpose, processing mechanism (Bouchacourt & Buschman, 2019; Swan & Wyble, 2014).

## Open practices statement

Experiments were preregistered on the Open Science Framework (OSF) repository (https://osf.io/h456p/). We preregistered ten experiments for this project, but for clarity and conciseness, only the most relevant experiments are reported in the article body. Information about remaining experiments is available on the OSF. Table 1 lists studies in chronological order, as well as OSF links and notes. All data and code are available on the OSF at https://osf.io/h456p/.

**Table 1:** Chronological order of experiments

Title	Open Science Framework (OSF) title and link	N	Format
**Experiment 1a**	**Does inter-item interference occur in feature-general or feature-specific codes? **(https://osf.io/tckms)	**40**	**In-lab**
S1	Retro-cue pilot (color) (https://osf.io/vsrxc)	25	Online
S2a	Retro-cue pilot (orientation) (https://osf.io/s7qrm)	25	Online
S2b	Retro-cue pilot (orientation) 2.0 (https://osf.io/dy6nj)	25	Online
S3a	Feature interference for shapes and colors (https://osf.io/df4z9)	30	Online
S3b	Feature interference for shapes and colors (https://osf.io/5n8pm)	30	Online
**Experiment 1b**	**Feature interference for colors and orientations** (https://osf.io/53rj4)	**30**	**Online**
S4	Mixed-category benefit: during encoding or maintenance? (https://osf.io/3efbq)	60	Online
S5	Mixed category benefit: pre-cue edition (https://osf.io/q8shb)	60	Online
**Experiment 2**	**Manipulating sensory encoding and memory contents simultaneously** (https://osf.io/6pbhu)	**40**	**Online**

Experiments shown in bold are included in the main article body; all others can be found in the Online Supplementary Material.

## Experiment 1a

### Method

#### Participants

We collected data from 44 participants from the University of California, San Diego (UCSD) community who completed the study for pay at a rate of $15/h or for course credit. Four participants met our preregistered exclusion criteria (see below), giving us a final total of 40 participants. Preregistered sample sizes for Experiment 1a, and all subsequent experiments, were chosen based on existing work in the literature. All participants were at least 18 years old, had normal or corrected-to-normal color vision, and reported no neurological disorders. All procedures were approved by UCSD’s Institutional Review Board.

#### Stimuli

The stimuli and experimental procedure were programmed using MATLAB and Psychophysics Toolbox 3 (Kleiner et al., 2007). Participants sat approximately 40 cm away from the computer display during the task. A chinrest was not used during the experiment, so all of the following visual angles are approximate. Stimuli were presented against a gray background with a fixation point that subtended 1 degree of visual angle. Color stimuli were circles 3^○^ in diameter, and on each trial colors were sampled uniformly from a 360° CIE L*a*b color space centered at *L* = 54, *a* = 18, and *b* = -8 (Adam et al., 2017). Monitors were not calibrated to render truly equiluminant colors, but as all manipulations were within-subjects, we do not believe that this produced systematic differences between experimental conditions. Oriented bars were dark rectangles 3^○^ in length and 1.05^○^ in width, and angles were sampled uniformly from a 180^○^ space.

**Fig. 1:**
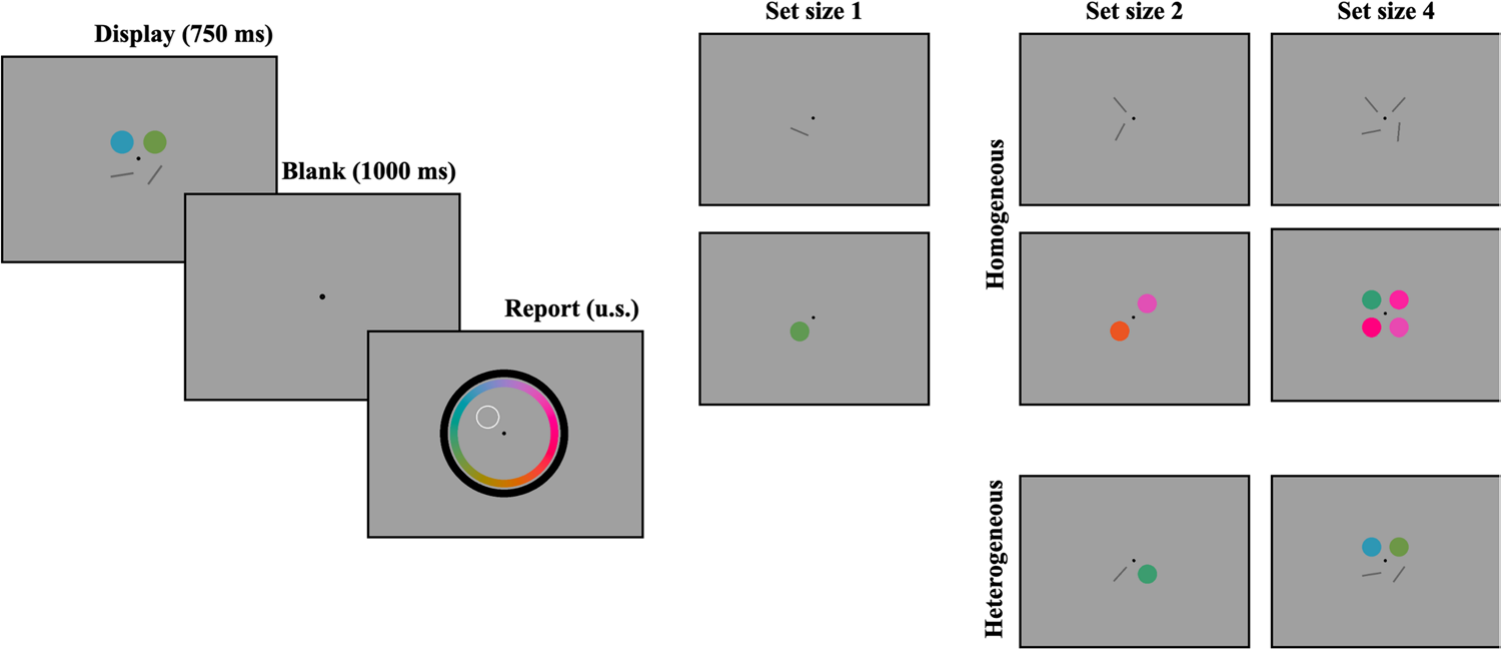
Procedure and conditions for Experiment 1a. Participants saw a display of objects, followed by a delay, and then an unspeeded report period (**left**). We used set sizes 1, 2, and 4, and set sizes 2 and 4 could be homogeneous or heterogeneous (**right**)

On each trial, up to four stimuli were presented at four equidistant, fixed locations around the screen, each 6^○^ away from the fixation point (see Fig. 1). On each trial, a subset of these locations was randomly selected (depending on trial set size). Stimuli appeared for 750 ms, followed by a blank delay of 1,000 ms, after which two continuous-report wheels appeared at fixed locations around the entire screen. The outer wheel had an outer radius of 16^○^, and the inner wheel had an inner radius of 13.5^○^. Both wheels had an arc thickness of 2^○^. Whether the color or orientation wheel appeared on the outside was randomly assigned to each subject. To be consistent with the orientation wheel, the position of each option on the color wheel remained constant across the experiment.

#### Procedure

The task (Fig. 1) was a continuous-report WM task (Wilken & Ma, 2004). At the start of each trial, one, two, or four items were presented on the screen. These items could be colors, oriented bars, or half colors and half oriented bars. Following the stimulus presentation and delay periods, one item from the display was probed for report by the item’s location on the screen, and participants had an unlimited amount of time to make a response. Participants made a response by clicking the location on the orientation or color wheel that matched the angle or color of the probed stimulus. Despite an orientation space of 180^○^, the orientation wheel was a complete circle, and participants were instructed that they could click either end of the wheel.

Trial set size (1, 2, or 4), display condition (homogeneous, heterogeneous), and probe feature (color, orientation) were fully counterbalanced, with one small exception: set size 1 trials had an undefined display condition, as they are neither homogeneous nor heterogeneous. These trials were coded as “homogeneous” in the task script but were not considered homogeneous for analysis purposes. Participants completed 75 trials per condition for a total of 750 trials across the ten conditions. These trials were spread out over 25 blocks of 30 trials each, and experimental conditions were fully counterbalanced within a block. Following each block, participants were given their average recall (in degrees), as well as the number of trials in which the feature category was incorrectly reported (e.g., participants reported an orientation when the probed stimulus was a color). Prior to the task, participants completed a set of ten practice trials, or one trial per experimental condition, and they received feedback after each trial.

#### Exclusion criteria

Based on pre-registered criteria, participants were excluded from all analyses if more than 10% of total trials were feature-report errors, or if any given condition had more than 20% feature errors (that is, reporting color when orientation was cued or vice versa). We preregistered these exclusion criteria to ensure that participants were attentive during the task and also to ensure that we obtained a sufficient number of usable trials, as we excluded all trials with feature report errors from our analyses. Previous work showing high accuracy in recalling feature categories (Awh et al., 2007; Scolari et al., 2008) suggests that these limits were not overly stringent. We also excluded a participant from all analyses if we lost more than 10% of data due to technical issues that occurred during the session (e.g., computer crashes). We preregistered that we would collect data until we had usable datasets from 40 participants. In Experiment 1a, we reached our sample size of 40 but excluded four participants who met the above criteria, so we continued data collection until we reached 40 usable datasets. In addition to those four subjects, we excluded 313 individual trials with feature report errors (1.04% of total trials).

#### Data analysis

We conducted all analyses using R, version 4.3.1 (R Core Team, 2023) and *tidyverse*, version 2.0.0 (Wickham, 2023). Data visualizations were created with the *ggplot2* package, version 3.4.3 (Wickham et al., 2023), as well as *viridis*, version 0.6.4 (Garnier et al., 2023).

Our primary interest was testing how heterogeneous displays affected the precision of WM. Because color and orientation have differently sized feature spaces (360^○^ and 180^○^, respectively), comparisons were conducted separately on each probed feature. For example, we compared trials with homogeneous orientation displays and trials with heterogeneous displays where an orientation was probed for report. Using the *circular* package, version 0.5–0 (Agostinelli & Lund, 2023), we computed the circular mean and standard deviation for each participant and experimental condition. Because orientation has a 180° space, we computed the circular standard deviation by multiplying the report error (in radians) for each trial by two, computing the circular standard deviation by condition, and then dividing the resulting standard deviation by two. We then ran a Bayesian two-way, repeated-measures ANOVA on the set size 2 and 4 conditions using *BayesFactor,* version 0.9.12–4.4 (Morey & Rouder, 2022) and default priors. We omitted the set size 1 conditions from this analysis because these conditions have an “undefined” display condition with respect to homogeneity of features, but we used these data in follow-up planned comparisons. To assess main effects of set size and display condition, we used Bayes factors to compare a full model with set size and display condition as fixed effects to reduced models with only one or the other. The Bayes factor ratio of the two competing models quantifies support for one model over the other, with Bayes Factors greater than 1 indicating relative support for the alternative model and Bayes factors less than 1 indicating relative support for the null model.

### Results

We found robust effects of set size and display condition but no interaction. Participants reported both colors and orientations with worse precision as set size increased, but precision was better for heterogeneous displays than homogeneous displays. Planned comparisons between our baseline set size 1 trials and higher set sizes revealed worse precision for heterogeneous and homogeneous display trials for both set size 2 and set size 4.

**Fig. 2:**
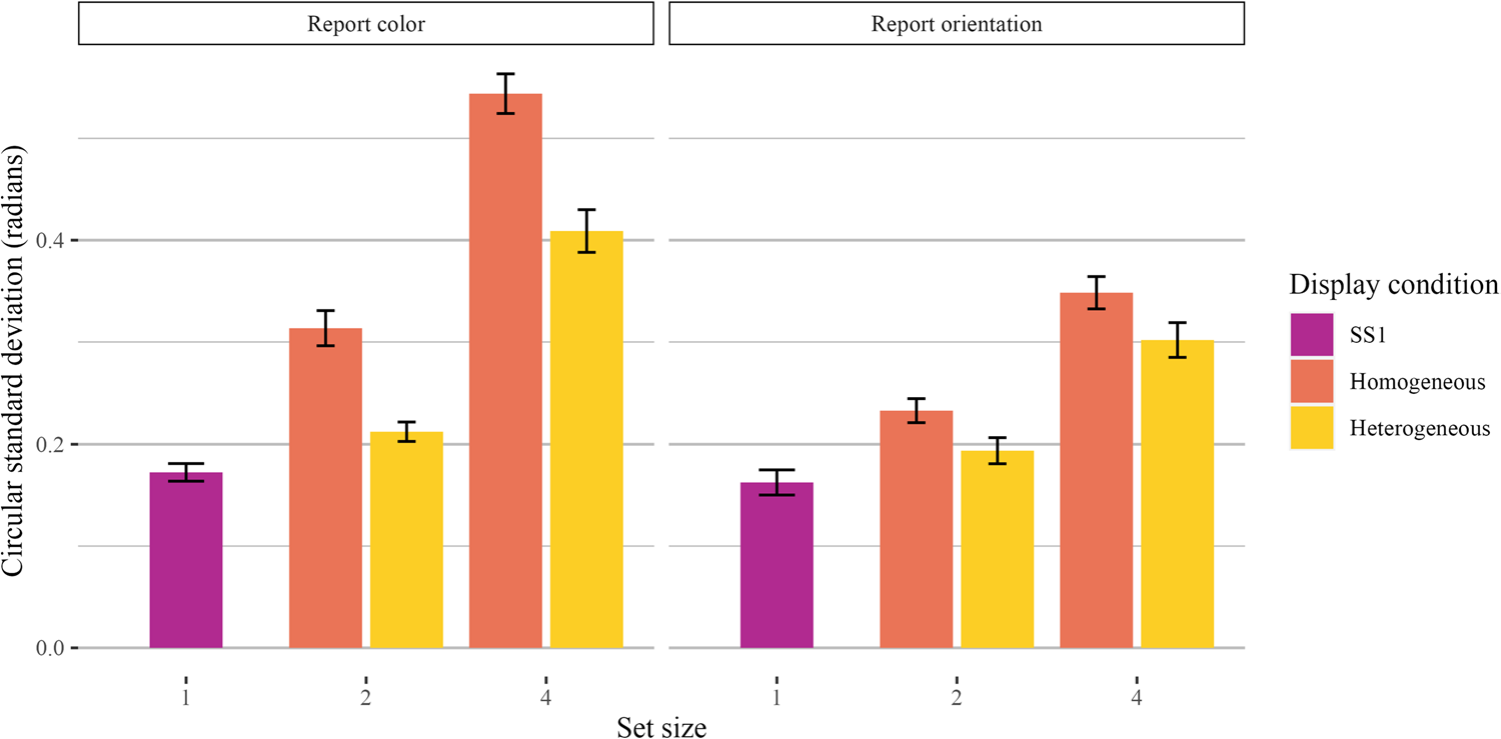
Main results of Experiment 1a. Results are shown separately for trials where participants reported color (**left**) and orientation (**right**). Bar plots quantify the circular standard deviation of the error distribution for each set size and display condition, and error bars represent the standard error of the mean

Supporting these conclusions, Bayes factor comparisons strongly preferred the expanded model over the model with display condition only (orientation: *BF*_*10*_ = 7.74 × 10^28^ ± 3.84%, $\eta$_*p*_^*2*^ = 0.70; color: *BF*_*10*_ = 2.67 × 10^30^ ± 1.75%, $\eta$_*p*_^*2*^ = 0.71). Set size 4 trials had worse precision (therefore, a higher circular standard deviation) than set size 2 trials. The full model with display condition was also strongly favorable (orientation: *BF*_*10*_ = 1.87 × 10^6^ ± 3.82%, $\eta$_*p*_^*2*^ = 0.25; color: *BF*_*10*_ = 5.95 × 10^12^ ± 3.62%, $\eta$_*p*_^*2*^ = 0.43). Participants had worse precision in their report of homogeneous trials than heterogeneous trials. Finally, we compared a model with set size, display condition, and an interaction between the two against a reduced model without the interaction. We saw weak evidence against an interaction between set size and display condition for orientation and equivocal evidence for color (orientation: *BF*_*10*_ = 0.25 ± 4.26%; color: *BF*_*10*_ = 0.51 ± 4.56%). A plot of the mean circular standard deviations is shown in Fig. 2.

Next, we conducted planned comparisons between our baseline set size 1 trials and higher set sizes. For color and orientation trials separately, we first compared the set size 1 trials to the set size 2 *homogeneous* trials. We found a main effect of set size (orientation: *BF*_*10*_ = 3.14 × 10^13^ ± 1.14%; color: *BF*_*10*_ = 4.11 × 10^9^ ± 2.98%). We also found a main effect of set size when we compared set size 1 and set size 2 *heterogeneous* trials (orientation: *BF*_*10*_ = 2.54 × 10^5^ ± 1.28%; color: *BF*_*10*_ = 4.76 × 10^4^ ± 1.85%). We also found a main effect of set size when comparing set size 1 trials to set size 4 homogeneous trials (orientation: *BF*_*10*_ = 2.57 × 10^17^ ± 1.36%; color: 2.13 × 10^28^ ± 0.88%) and heterogeneous trials (orientation: *BF*_*10*_ = 1.33 × 10^15^ ± 2.05%; color: 2.87 × 10^16^ ± 0.94%).

#### Post hoc swap analyses

Our manipulation of display homogeneity raises the question of demands required to bind the properties of an object to its specific spatial or temporal context (Oberauer & Lin, 2017). Previous work claims that homogeneous displays place stronger demands on context binding, leading to higher competition at the report stage and an increased likelihood of inter-item swaps (Cai et al., 2020, 2022). Thus, it is possible that our results are driven by context binding demands rather than feature-specific competition. Relatedly, displays with two colors, for example, may have lower precision than displays with one color and one orientation because participants are more likely to swap the two colors than they are the color and orientation (Awh et al., 2007). Thus, to evaluate the impact of within-category swap errors, we took all set size 2, homogeneous trials (e.g., trials with two colors or two orientations) and computed the response error with respect to the probed item (e.g., report error) and the response error with respect to the *unprobed* item. A low response error with respect to the unprobed item is associated with a higher likelihood that the participant swapped the two items (e.g., if error with respect to the unprobed item is close to zero, it is possible that participants instead reported the color or orientation of the unprobed item). This analysis was post hoc and, therefore, not pre-registered.

**Fig. 3:**
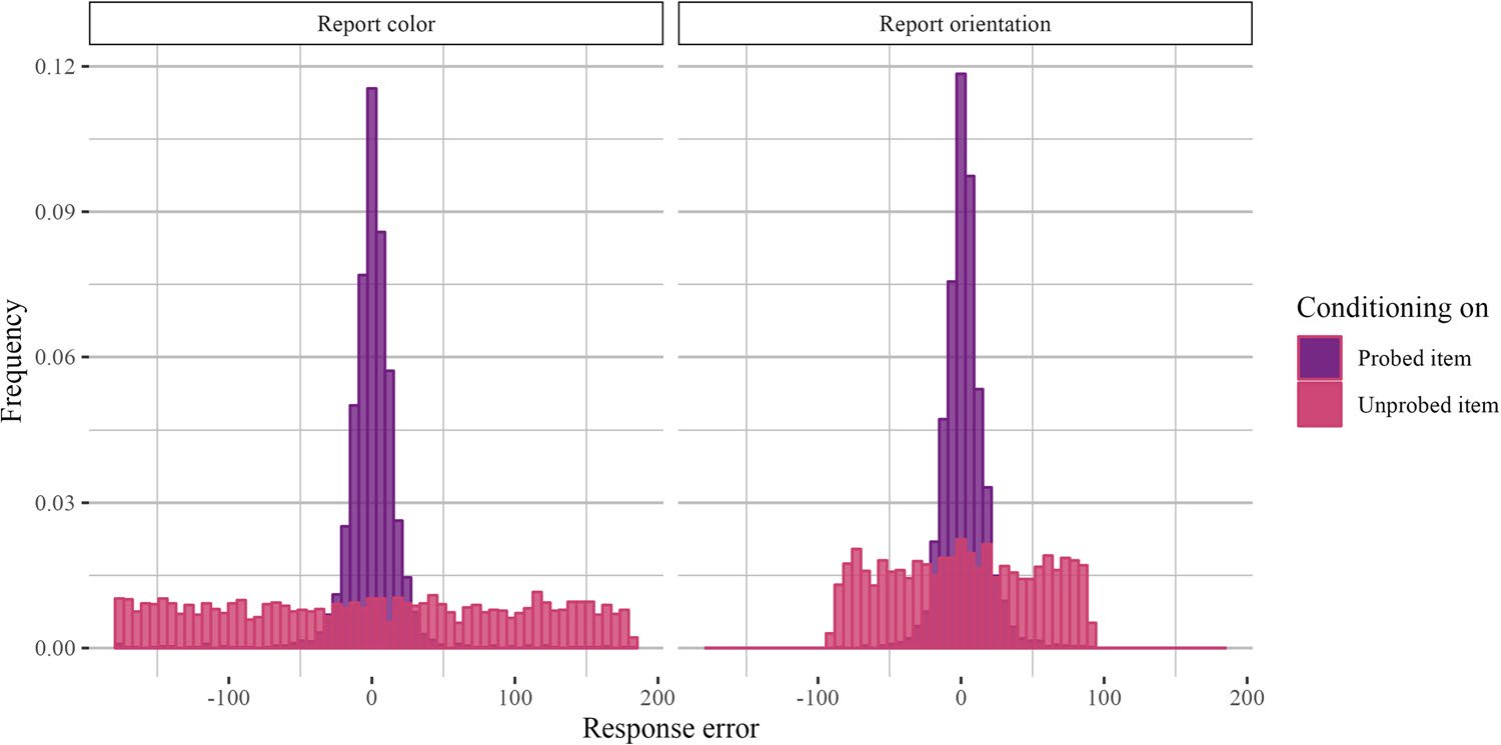
Histograms of response errors for set size 2, homogeneous trials (Experiment 1a). Response error plotted with respect to the probed item and with respect to the unprobed item

Figure 3 shows histograms of response errors with respect to the probed and unprobed items. We used an information theoretic approach to assess uniformity of the response distribution with respect to the unprobed items (Panichello et al., 2019). Shannon Entropy is maximized for uniform distributions, so we compared the entropy of response distributions with respect to the unprobed item to the distributions of the unprobed item angles, which were drawn from a circular uniform distribution. This information-theoretic measure makes fewer assumptions than other models (e.g., approaches based on a mixture model or a signal detection model), as it simply assesses whether overall entropy is lower than might be expected from the actual distribution of feature values used in the experiment (i.e., whether there is clustering in the response error). We obtained by-participant differences in Shannon entropy for the observed and expected distributions and ran a Bayesian *t-*test to assess whether the mean difference is greater than zero. The test favored the null hypothesis of no mean difference in entropy (orientation: *BF*_*10*_ = 0.17 ± 0.05%; color: *BF*_*10*_ = 0.33 ± 0.04%). We also obtained posterior samples for the mean difference in entropy over 6,000 iterations and found that the 95% posterior density interval contained zero for color and orientation reports (orientation: [-0.0167, 0.0168], color: [-0.00510, 0.0190]). These analyses suggest that the response distribution with respect to the unprobed item is relatively uniform and that context binding errors or swapping alone cannot explain our findings.

## Experiment 1b

In Experiment 1b, we replicated the main finding of Experiment 1a using a web-based study and a different group of participants.

### Method

#### Participants

We used Prolific to recruit 40 participants living in the USA. All were at least 18 years old and had normal or corrected-to-normal color vision with no color blindness. Prior to beginning the experiment, all participants gave informed consent. Procedures were approved by UCSD’s Institutional Review Board.

#### Stimuli

We used jsPsych, version 7 (de Leeuw & Gilbert, 2023) to create the stimuli and experimental procedure, and participant data were uploaded to a secure server as a JSON file. Participants were required to complete the experiment on a desktop computer (as opposed to a smartphone or tablet), but they sat at unknown distances from the display.

Colors and orientations were chosen randomly from 360^○^ and 180^○^ spaces, respectively, with the constraint that colors and orientations appearing in the same trial were at least 15^○^ apart in circular space, after Schurgin et al. (2020). Due to variation in luminance and display settings across personal computers, color stimuli may have varied across participants. While this produced a source of variance across participants, all experimental manipulations were within-subjects.

#### Procedure

**Fig. 4:**
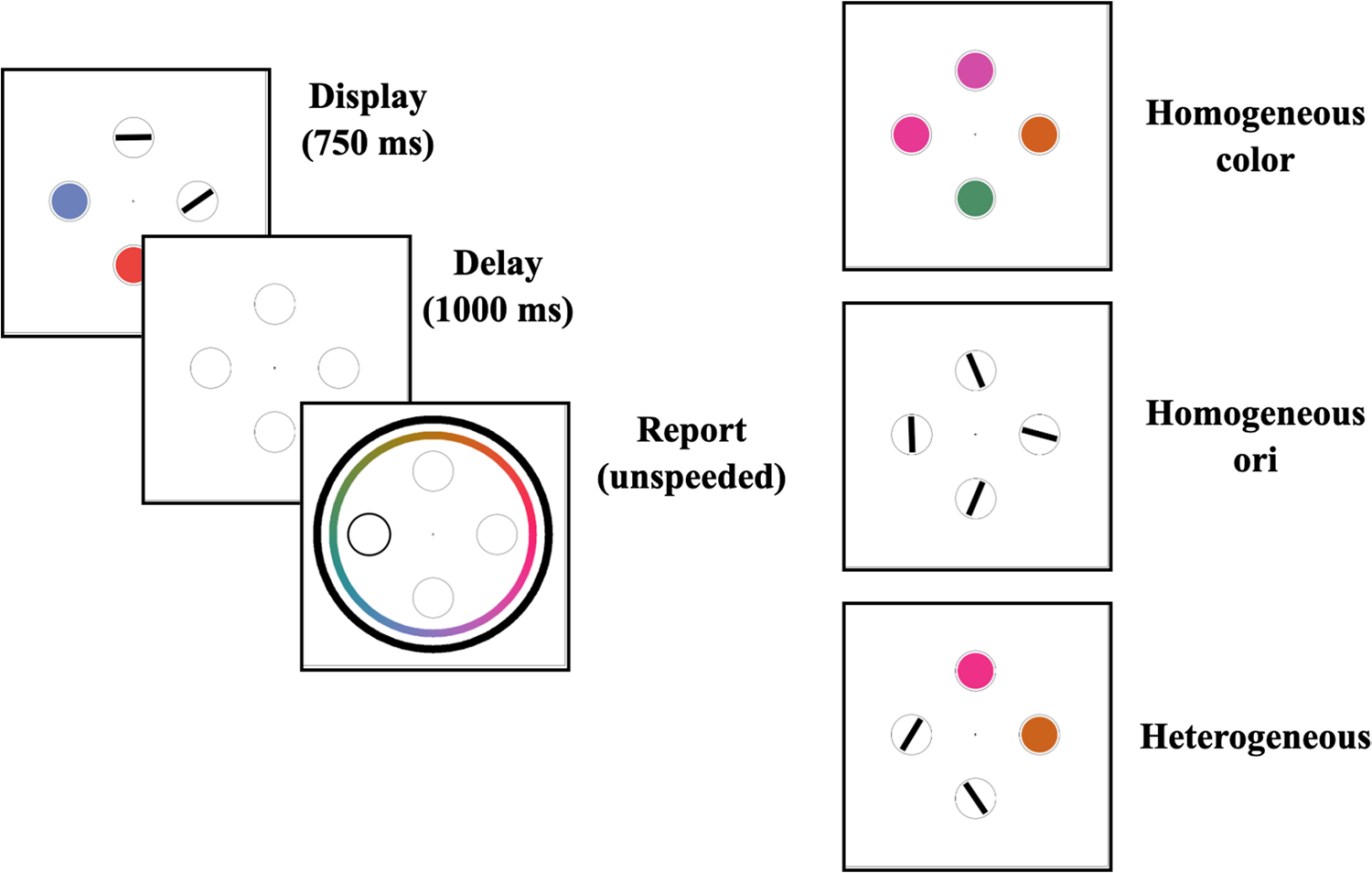
Procedure and conditions for Experiment 1b. Trial structure (**left**) and example displays for homogeneous colors (**top**), homogeneous orientations (**middle**), and heterogeneous displays with two each of colors and orientations (**bottom**)

A diagram of the trial structure is shown in Fig. 4. Participants clicked a central fixation cross to begin the trial. Following each click, there was a 1,500-ms delay followed by the presentation of four stimuli for 750 ms. Experimental conditions were balanced identically to set size 4 trials in Experiment 1a. After the offset of the stimuli, there was a 1,000-ms delay, during which the placeholder circles were present but the screen was otherwise blank. At the onset of the report window, a color wheel and an orientation report wheel appeared around the placeholder circles, and the placeholder circle in the probed location had a darker border. Trials were counterbalanced so that when a heterogeneous display was shown, participants were probed to report a location with a color on half of trials and a location with an orientation on the other half. As participants moved their cursor around the report wheels, the probed location filled in with the color or orientation corresponding to their cursor’s position on the wheel. Participants had unlimited time to click a location on the wheel, which locked in their response, concluding the trial. After every trial, participants were given feedback about their error in degrees, as well as feedback if they clicked the incorrect wheel.

There were 20 practice trials followed by 300 main task trials, giving 75 main task trials in each of the four experimental conditions (homogeneous colors, homogeneous orientations, heterogeneous display with a color report, heterogeneous display with an orientation report).

#### Exclusion criteria

Participants who clicked the incorrect report wheel on more than 20% of trials in any of the four conditions were excluded from all analyses, and for all participants we excluded individual trials with an incorrect feature report. No participants were excluded, but we removed 132 individual trials where the incorrect feature wheel was clicked (1.1% of trials).

#### Data analysis

We parsed JSON files using the *jsonlite* package in R, version 1.8.7 (Ooms, 2014), but data processing and aggregating methods were the same as Experiment 1a. We also followed the same procedure for Bayesian inference.

### Results

**Fig. 5:**
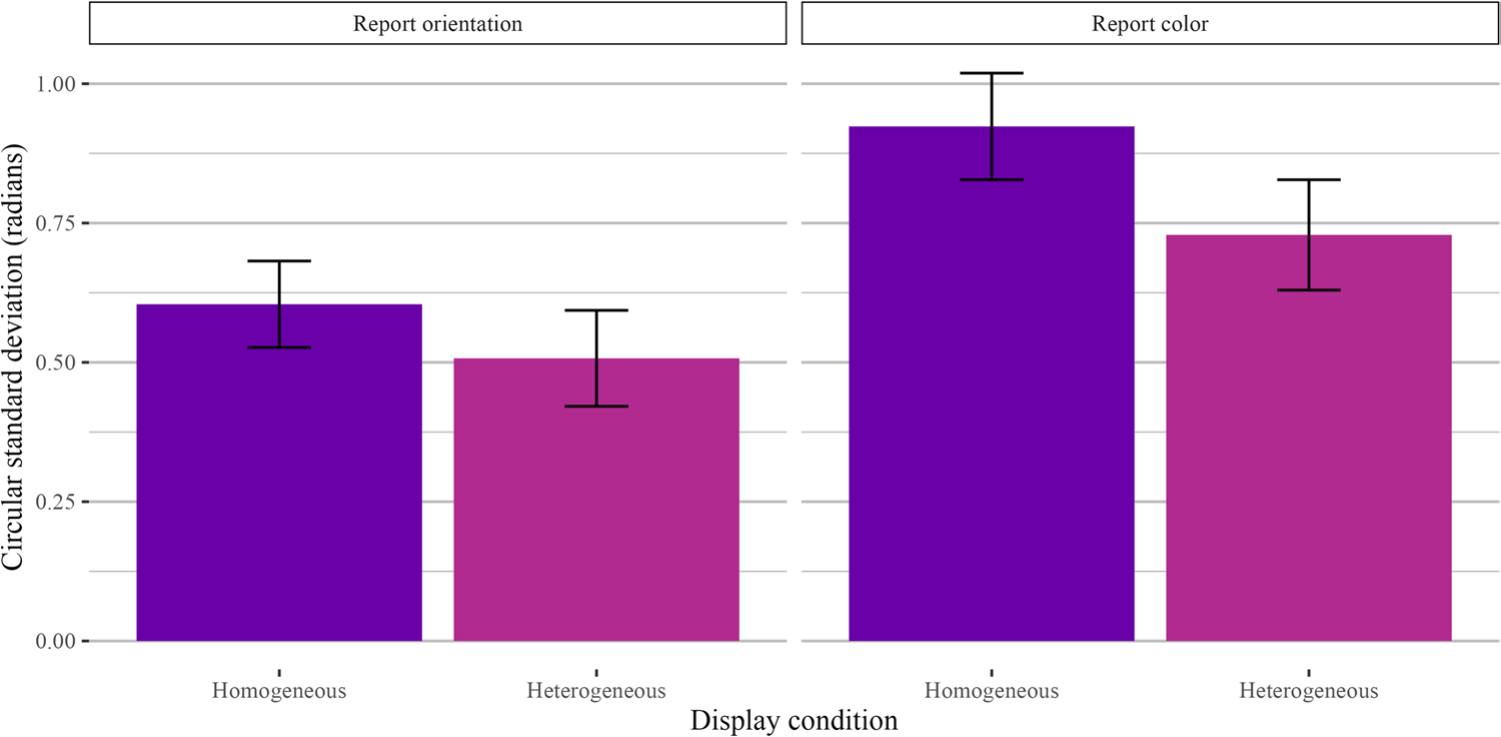
Main results for Experiment 1b. Results are shown separately for trials where participants reported orientation (**left**) and color (**right**)

The results of Experiment 1b directly replicated Experiment 1a at set size 4, with performance significantly better for heterogeneous displays than for homogeneous displays. Performance in each condition is shown in Fig. 5. We ran a Bayesian two-way, repeated-measures ANOVA on color probe and orientation probe trials separately, with display condition (homogeneous vs. heterogeneous) as the fixed effect and participant as the random effect. Bayes factor comparisons strongly preferred the model with display condition over the intercept-only model (orientation: *BF*_*10*_ = 19.6 ± 0.83%, Cohen’s *d* = 0.64; color: *BF*_*10*_ = 1.59 × 10^4^ ± 0.94%, Cohen’s *d* = 0.99).

### Discussion

In Experiment 1a, increasing set size impaired precision with both homogeneous and heterogeneous displays, in line with previous findings (Bays et al., 2009; Ma et al., 2014). However, performance was significantly better for heterogeneous displays than homogeneous displays, suggesting at least some role of feature-specific interference. We then performed an entropy-based swap analysis to assess the possibility that our results are driven instead by context-binding demands as opposed to inter-item interference (Cai et al., 2022). In Experiment 1b, we used a web-based study to replicate Experiment 1a at set size 4, and precision was higher for heterogeneous displays than for homogeneous displays. The results add further evidence for feature-specific interference and validate the use of jsPsych and Prolific in Experiment 2.

Despite clear evidence that performance is better with heterogeneous displays, the mechanism of this benefit is unknown. While heterogeneous displays may reduce inter-item competition during maintenance, these data could also be explained by feature-similarity based competition during encoding. In Experiment 2, we used a retro-cue design (Nobre et al., 2004; Souza & Oberauer, 2016), which allowed us to manipulate the heterogeneity of the display (thereby assessing the role of similarity during encoding), as well as the heterogeneity of retro-cued items (thereby assessing the role of similarity during maintenance).

## Experiment 2

### Method

#### Participants

We used Prolific to recruit 44 participants living in the USA. Screening criteria and informed consent procedures were the same as in Experiment 1b. All participants completed both sessions of the experiment.

#### Stimuli

The stimuli were identical to those used in Experiment 1b except where noted below.

#### Procedure

**Fig. 6:**
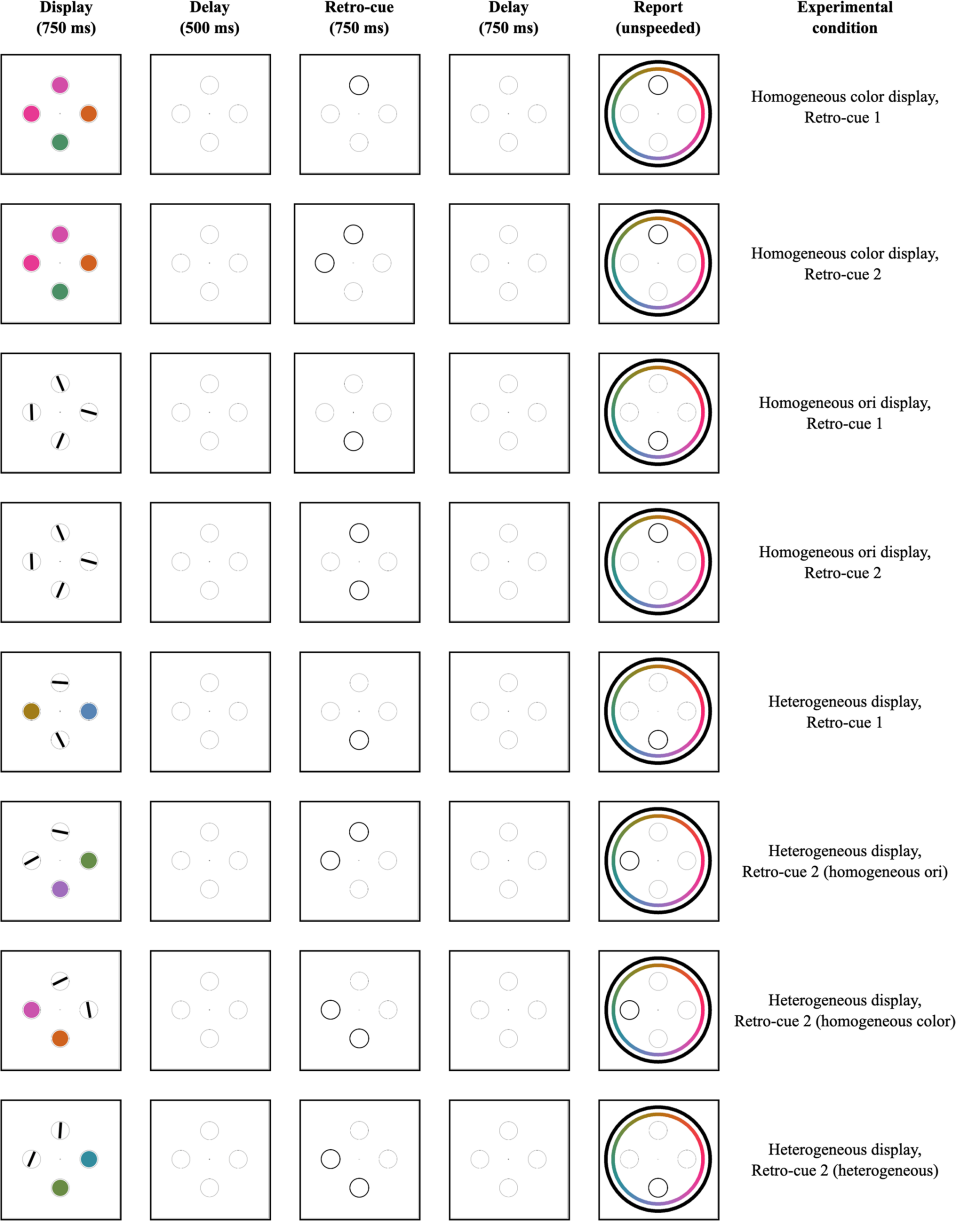
Example displays for Experiment 2. This diagram omits conditions that differed only in the feature probed for report (e.g., heterogeneous displays where a color and orientation are retro-cued)

A diagram of the trial structure and experimental conditions is given in Fig. 6. Clicking a central fixation cross initiated the start of the trial after a 1,500-ms delay. The displays consisted of four colors on 25% of trials, four orientations on 25% of trials, and two each of colors and orientations on 50% of trials. The stimuli were present for 750 ms, followed by a 500-ms blank delay. Next, one or two of the placeholder circles had a darker border for 750 ms, indicating which item, or items, could be probed later. The retro-cue circles disappeared for 750 ms before the unspeeded report. At the onset of the report window, a color wheel and an orientation report wheel appeared, and the placeholder circle in the probed location had a darker border. Participants made only one report per trial, and the probed location was always one that was cued during the delay period.

Participants completed two sessions of equal length, and experimental conditions were counterbalanced within a session. We manipulated the display condition (homogeneous display, heterogeneous display), the feature ultimately probed for report (color, orientation), the retro-cue set size (one item, two items), and the retro-cue condition (homogeneous items retro-cued, heterogeneous items retro-cued). In total, this design produced ten experimental conditions. Experimental conditions occurred equally often over the course of the experiment, with the exception that participants completed twice as many trials with a homogeneous display of items and two items retro-cued. Although this created an imbalance in the number of trials per condition, it ensured equal numbers of trials with homogeneous and heterogeneous displays, and equal relative frequencies of retro-cue set sizes (one item cued vs. two items cued) across homogeneous and heterogeneous display conditions. Procedures for reporting were identical to Experiment 1a.

Participants completed a set of 12 practice trials, and the frequency of experimental conditions mirrored those used in the main task. There were 360 main task trials per session, giving a total of 24 practice trials and 720 main task trials. The two conditions with a homogeneous display condition and two items retro-cued had twice as many trials as other conditions, giving 120 trials in each of those two conditions and 60 trials in each of the other conditions.

#### Exclusion criteria and sequential data collection

We preregistered a final sample size of 40 usable participants. Because the interpretability of our experiments rests on participants using the retro-cue as intended, we preregistered a sequential data collection process to avoid wasting time and resources. The retro-cue effect is widely observed in cognitive psychology and neuroscience research (Souza & Oberauer, 2016), and the presence of a retro-cue effect in homogeneous display conditions served as a positive control. After 20 participants, we compared one-item and two-item retro-cue conditions for homogeneous trials and performed no additional analyses. After observing a numerical retro-cue effect for both color and orientation reports, we collected data from the remaining participants. Had we not observed a numerical effect, we would have discontinued data collection, adjusted experimental parameters, updated our preregistration, and started data collection over. Our exclusion criteria were the same as Experiment 1b. At the end of data collection, we excluded four participants from all analyses and 496 individual trials (or 1.7% of trials).

#### Data analysis

We processed, aggregated, and analyzed the data using the same methods as Experiment 1.

### Results

#### Set size

**Fig. 7:**
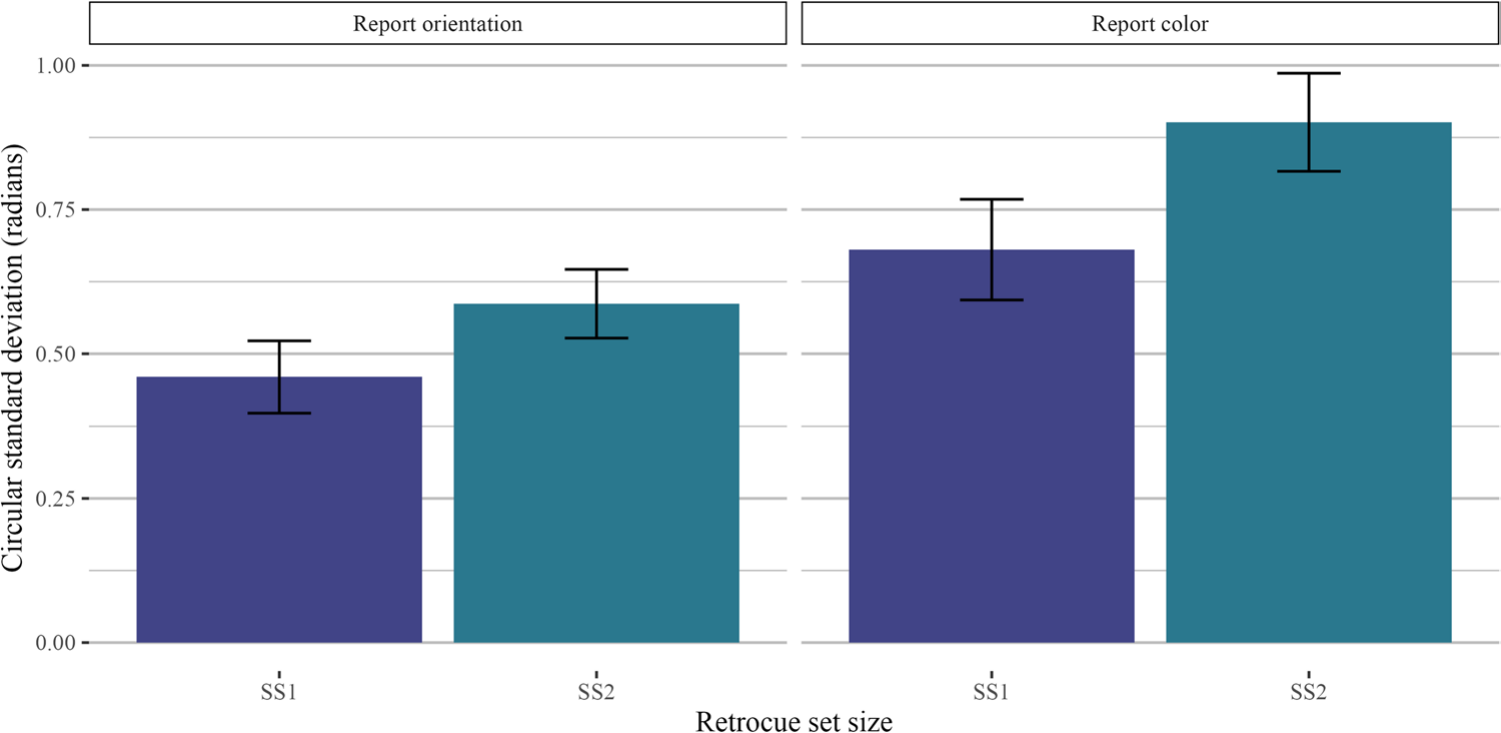
Results for Experiment 2 homogeneous trials. Results are shown separately for trials where participants reported orientation (**left**) and color (**right**)

Our first comparison of interest was to look at the effect of retro-cue set size (one item vs. two items retro-cued) as a positive control, as the interpretability of subsequent analyses hinges on the assumption that participants were using the retro-cue as intended (i.e., that they were not simply holding all four items in mind on every trial). As hypothesized, performance was better when one item was retro-cued than when two items were retro-cued. Thus, when comparing trials across different retro-cue conditions, null effects are unlikely the result of non-compliance with experiment instructions. We filtered the data to include only homogeneous display trials and ran a two-way Bayesian repeated-measures ANOVA with the retro-cue set size as a fixed effect and participant ID as a random effect. Model comparisons strongly preferred the full model over the intercept-only model (orientation: *BF*_*10*_ = 3.97 × 10^7^ ± 1.07%, *d* = 1.37; color: *BF*_*10*_ = 2.39 × 10^7^ ± 1.18%, *d* = 1.34). A plot of the circular standard deviations is shown in Fig. 7.

#### Mixed category benefit during encoding

**Fig. 8:**
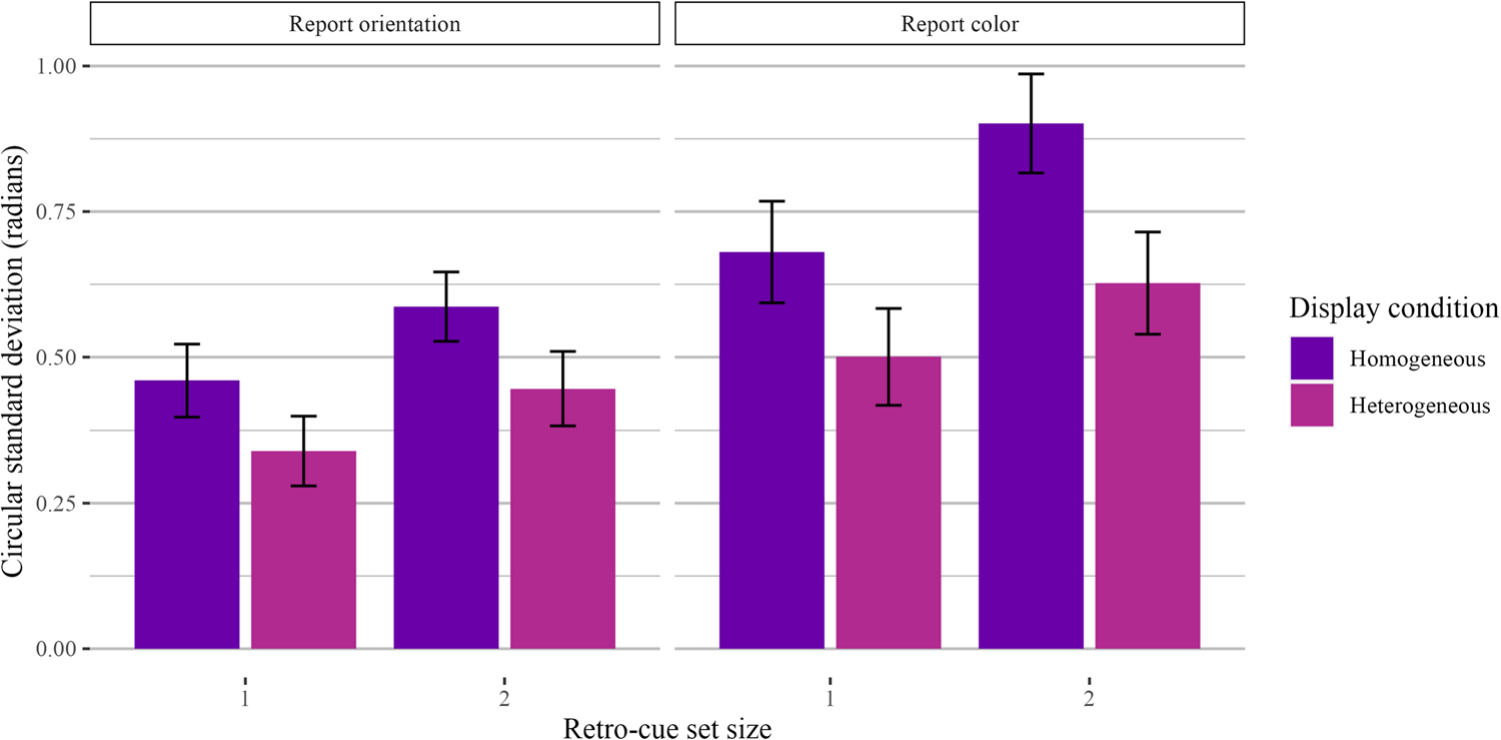
Results for Experiment 2 trials with set size 1 or homogeneous retro-cue conditions. In this visualization, we kept retro-cue condition constant (retro-cue only colors, or only orientations) and visualized display condition. Results are shown separately for trials where participants reported orientation (**left**) and color (**right**)

The following analysis was mistakenly omitted from the preregistration document. To assess the mixed category benefit during encoding, we took trials with a homogeneous or SS1 *retro-cue* condition, or trials where one item was retro-cued or two items of the same feature were retro-cued. Using color trials as an example, the display condition was either four colors or two colors and two orientations, but we filtered data to include only trials with two colors retro-cued. We then compared performance across set sizes and display conditions. Performance was better when one item was retro-cued compared to two items, and performance was better when the display condition was heterogeneous compared to homogeneous. In other words, even when participants ultimately maintained homogeneous sets of items in WM, performance was better when the display condition was heterogeneous, replicating Experiments 1a and 1b. The results are plotted in Fig. 8. We performed these analyses with a two-way Bayesian repeated-measures ANOVA with display condition and set size as fixed effects and participant ID as a random effect. For both color and orientation, Bayes factor comparisons strongly favored the full model with both set size and display condition. There was a strong main effect of set size (orientation: *BF*_*10*_ = 5.53 × 10^16^ ± 2.67%, $\eta$_*p*_^2^ = 0.51; color: BF_10_ = 3.10 × 10^11^ ± 4.00%, $\eta$_*p*_^2^ = 0.40) and display condition (orientation: BF_10_ = 5.94 × 10^19^ ± 3.48%, $\eta$_*p*_^2^ = 0.57; color: BF_10_ = 4.75 × 10^17^ ± 4.09%, $\eta$_*p*_^2^ = 0.53).

#### Mixed category benefit during maintenance

**Fig. 9:**
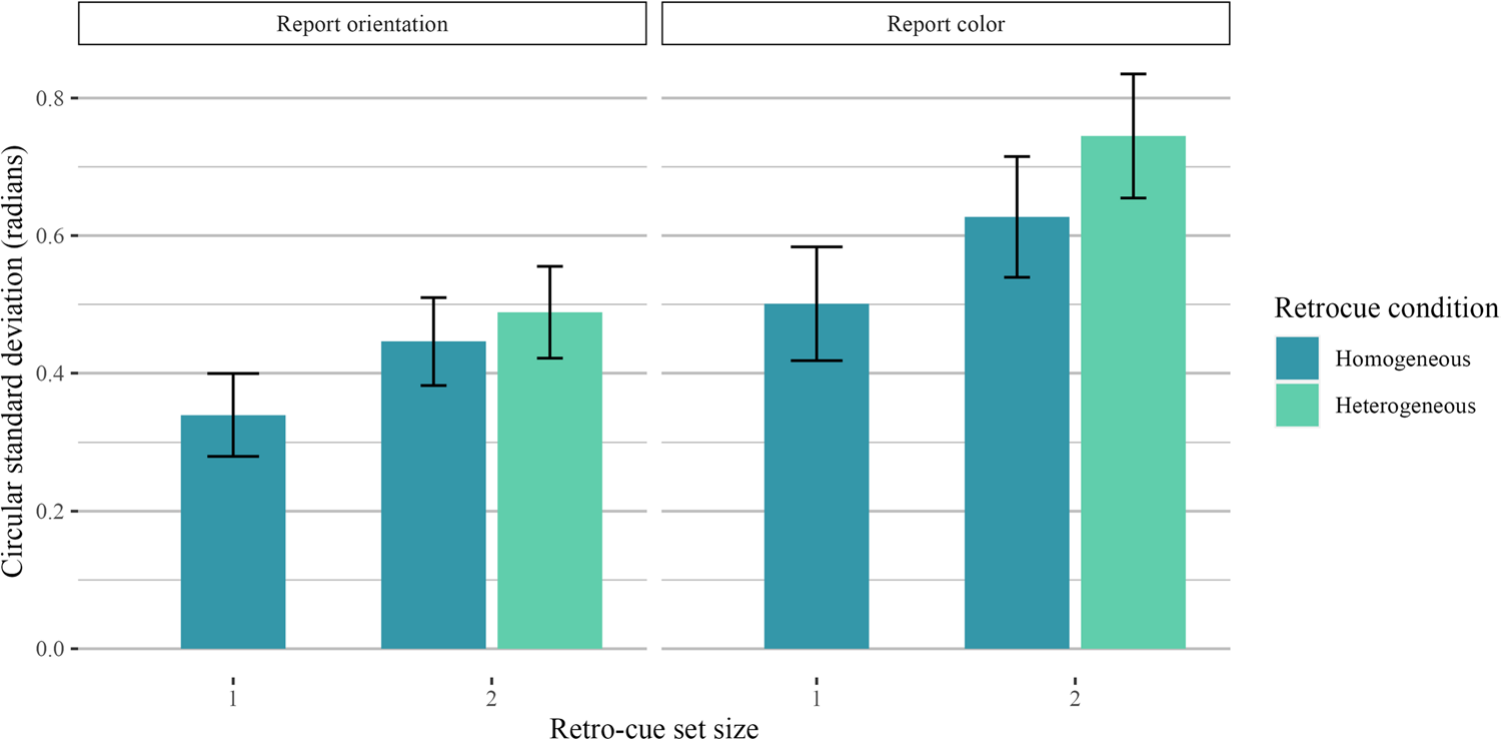
Results for Experiment 2 trials with heterogeneous displays. In this visualization, we kept display condition constant (two colors and two orientations) and visualized retro-cue condition. Results are shown separately for trials where participants reported color (**left**) and orientation (**right**)

Our final analysis kept display (and, thus, encoding) conditions constant and compared performance across different retro-cue conditions (see Fig. 9). We only analyzed trials with a retro-cue set size of 2 and a heterogeneous display condition, and we compared trials with homogeneous retro-cues (i.e., two of the same feature) and heterogeneous retro-cues (i.e., one color and one orientation). For color-report trials, performance was better for *homogeneous* retro-cues than for heterogeneous retro-cues. For orientation-report trials, performance was numerically better for homogeneous retro-cues, but the Bayes factor comparison was equivocal. Regardless, performance differences in both color and orientation trials provided no evidence for feature-specific interference when retro-cues were involved and the properties of the stimuli during encoding were controlled. For color-report trials, model comparisons favored the full model with retro-cue condition as a fixed effect (*BF*_*10*_ = 2.70 × 10^3^ ± 1.21%, *d* = 0.84), though performance was better for homogeneous retro-cues. For orientation-report trials, Bayes factor comparisons also favored the full model with retro-cue condition as a fixed effect, albeit very weakly (*BF*_*10*_ = 3.58 ± 0.71%, *d* = 0.42).

### Discussion

In Experiment 2, we observed feature-specific interference during encoding, consistent with Experiments 1a and 1b. However, the previously observed performance benefits for heterogeneous sets of items disappeared when these items were retro-cued. In other words, when participants encoded a heterogeneous display, and we compared performance when two colors or two orientations were retro-cued or one of each feature was retro-cued, performance was better for two colors. Further, the null findings were unlikely a result of non-compliance with experiment instructions, as performance was better when one item was retro-cued than when two items were retro-cued.

## General discussion

The goal of the present work was to manipulate display homogeneity to test feature-specific or feature-general sources of interference in WM (i.e., interference within and across feature spaces). In Experiment 1a, increasing the display set size produced a cost in mnemonic precision regardless of whether the displays were homogeneous or heterogeneous. However, when controlling for set size, mnemonic precision was better for heterogeneous displays compared to homogeneous displays. These findings replicate and extend previous research on the mixed-category benefit (Avital-Cohen & Gronau, 2021; Cohen et al., 2014). More importantly, these results suggest that inter-item competition occurs in both a feature-general manner as more items are remembered, and in a feature-specific manner that depends on item similarity. In Experiment 1b, we replicated the findings of Experiment 1a at set size four and validated the use of online experiments for these studies more generally. Experiment 2 used a retro-cueing design and revealed that encoding a display of heterogeneous items is advantageous for mnemonic precision but that once a given set of items are encoded into WM, the feature-specific interference disappears and there is no benefit associated with remembering heterogeneous sets (with mild evidence that homogeneous displays are remembered with higher precision). Thus, feature-specific interference likely arises during sensory encoding, but once encoded, there is no evidence for feature-specific competition during maintenance in WM.

One key motivation for our experiment is that different instantiations of sensory recruitment models of WM make qualitatively different predictions about the role of feature similarity in inter-item interference. For example, some models assume that inter-item competition occurs via competition in higher-order processing stages that aggregate information from many feature-selective sensory neurons tuned to different features in earlier processing stages (Bouchacourt & Buschman, 2019; Swan & Wyble, 2014). In terms of behavior, increasing the set size should reduce WM performance because of more convergent input to high-order areas, but the combinations of feature types should not matter. Overall, the results of Experiment 2 indicate that once items are encoded into WM, the nature of the inputs matters little – a finding consistent with this class of model.

We were somewhat surprised to find that for Experiment 2, retro-cued homogeneous items were remembered with numerically better precision than retro-cued heterogeneous items, though this effect was weak for reported orientations. Nevertheless, this finding is consistent with Bouchacourt and Buschman (2019), where increasing inter-item similarity increases lateral excitatory connections between like-tuned units in the sensory layers, improving the stability of representations in WM. This dovetails with empirical work where increased similarity improved performance on a change-detection task (Lin & Luck, 2009), as well as where inter-item similarity facilitates internal selection (Kiyonaga & Egner, 2013). Perhaps selecting heterogeneous items internally from WM – as is required by a retro-cue task – comes at a higher cost than selecting more similar, homogeneous items. Nevertheless, further research should rigorously test a possible cause for this boost in performance.

Our experimental work supports the existence of coordinated communication between highly specialized and highly flexible populations of neurons, a framework with strong connections to recent theoretical perspectives such as priority maps and object files. For example, the feature-general “random layer” of Bouchacourt and Buschman (2019) integrates information from topographically organized layers that functionally resemble priority maps observed in early visual cortex (Mazer & Gallant, 2003; Treisman, 1986, 1988; Treisman & Gelade, 1980) and in intraparietal sulcus (Bisley & Goldberg, 2003; Bisley & Mirpour, 2019; Serences & Yantis, 2006; Sprague & Serences, 2013; Sprague et al., 2018). In turn, neurons in the random-layer exhibit high-dimensional tuning functions consistent with flexibly tuned neurons such as those frequently observed in prefrontal cortex (Fusi et al., 2016; Mante et al., 2013; Rigotti et al., 2013). Relatedly, data from Experiment 2 are generally consistent with the idea that content-independent pointers support the maintenance of information in WM in the form of “object files” after these items are encoded (Luria & Vogel, 2011; Ngiam et al., 2024; Pylyshyn, 1989; Thyer et al., 2022).

The finding that memory representations are robust to feature-specific interference suggests a prominent role of higher-order regions in mediating inter-item competition. However, our data are agnostic about whether item-specific information is stored in a sensory-like code (Iamshchinina et al., 2021) or whether it is re-coded into a non-sensory format and stored in higher-order brain areas (Xu, 2020). For example, Bouchacourt and Buschman (2019) proposed that inter-item interference originates due to destructive interference in higher layers where units receive convergent inputs from many sensory neurons with different feature-specific tuning functions (Swan & Wyble, 2014). However, the disruption of memory representations is realized via the backpropagation of signals from higher layers to the sensory layers where information about each remembered item is actually maintained. Thus our observation that competition does not have a strong feature-selective component is consistent with prior work demonstrating that high-fidelity mnemonic information is maintained in sensory cortices (Harrison & Tong, 2009; Rademaker et al., 2019; Serences et al., 2009). Equally, our results could be accommodated by models in which sensory regions are active during encoding, but activity in higher-order areas forms the basis for maintaining active memory representations and behavioral read-out (Bettencourt & Xu, 2016; Xu, 2020). The behavioral data presented here cannot adjudicate between these two models of storage without further constraints provided by neural data.

In sum, our data suggest that inter-item interference is feature-specific during sensory encoding but feature-general once items are in WM. These results are consistent with theoretical accounts of WM in which populations of unspecialized neurons in higher-order brain regions aggregate information from sensory-tuned neural populations early in visual processing. More broadly, we provide empirical support for the hypothesis that coordinated communication between highly-specialized and highly-flexible neurons gives rise to WM’s flexible and adaptive nature.

## Data Availability

All data are available on the Open Science Framework (see Open Practices Statement in article).

## Code availability

All code is available on the Open Science Framework (see Open Practices Statement in article).
